# Ventilation distribution in rats: Part 2 – A comparison of electrical impedance tomography and hyperpolarised helium magnetic resonance imaging

**DOI:** 10.1186/1475-925X-11-68

**Published:** 2012-09-11

**Authors:** Kimble R Dunster, Marlies EJ Friese, John F Fraser, Graham J Galloway, Gary J Cowin, Andreas Schibler

**Affiliations:** 1 Paediatric Critical Care Research Group, Paediatric Intensive Care Unit, Mater Children’s Hospital, South Brisbane, 4101, QLD, Australia; 2 Critical Care Research Group, The Prince Charles Hospital, Brisbane, QLD, Australia; 3 Centre for Advanced Imaging, The University of Queensland, Brisbane, QLD, Australia; 4 Medical Engineering, Queensland University of Technology, Brisbane, QLD, Australia

**Keywords:** Electrical impedance tomography, Hyperpolarized helium magnetic resonance lmaging, Ventilation distribution, Functional lung imaging

## Abstract

**Background:**

Hyperpolarised helium MRI (He3 MRI) is a new technique that enables imaging of the air distribution within the lungs. This allows accurate determination of the ventilation distribution *in vivo*. The technique has the disadvantages of requiring an expensive helium isotope, complex apparatus and moving the patient to a compatible MRI scanner. Electrical impedance tomography (EIT) a non-invasive bedside technique that allows constant monitoring of lung impedance, which is dependent on changes in air space capacity in the lung. We have used He3MRI measurements of ventilation distribution as the gold standard for assessment of EIT.

**Methods:**

Seven rats were ventilated in supine, prone, left and right lateral position with 70% helium/30% oxygen for EIT measurements and pure helium for He3 MRI. The same ventilator and settings were used for both measurements. Image dimensions, geometric centre and global in homogeneity index were calculated.

**Results:**

EIT images were smaller and of lower resolution and contained less anatomical detail than those from He3 MRI. However, both methods could measure positional induced changes in lung ventilation, as assessed by the geometric centre. The global in homogeneity index were comparable between the techniques.

**Conclusion:**

EIT is a suitable technique for monitoring ventilation distribution and inhomgeneity as assessed by comparison with He3 MRI.

## Introduction

Ventilation in homogeneity of the lung currently can be measured either with inert gas techniques or with imaging techniques using a radio-labeled or other tracer gas. [1-3]. Multiple breath washout techniques can measure global ventilation homogeneity, but cannot identify regional ventilation distribution [4]. In contrast, MRI using hyperpolarised helium-3 MRI (He3 MRI) gives true anatomic images of ventilation distribution. These measurement and imaging techniques all use tracer gases with density and viscosity different to air. [5] In Part 1 we investigated the impact of gas density on regional ventilation distribution using electrical impedance tomography (EIT Part I) and demonstrated that regional ventilation distribution was independent of gas density [6].

EIT assesses regional ventilation distribution by measuring transthoracic impedance changes during breathing and constructing images based, primarily, on the change of air content within the chest [7]. As the images are based on changes in air content, they are functional images, rather than anatomic images.

EIT has advantages over He3 MRI in clinical settings as the patient does not need to be moved to the He3 MRI scanner and routine ventilation can continue uninterrupted. Helium-3 is costly and in short supply worldwide, and polarization requires considerable time using elaborate and costly apparatus. Once polarized, the gas cannot be stored for long periods reducing the ready availability of the technique at critical times. With He3 MRI having a higher spatial resolution than EIT, it may provide more detailed regional ventilation distribution data. In contrast, EIT can be readily applied at the bedside in any clinical scenario. Both He3 MRI and EIT can measure regional ventilation distribution and global ventilation in homogeneity. As such, it is necessary to know if EIT can serve as a surrogate measure for the analysis of ventilation distribution, especially in patients who cannot be transported to a He3 MRI scanner.

This study compares measures of ventilation distribution and ventilation in homogeneity obtained from the anatomic images of He3 MRI and the functional images of EIT in ventilated rats.

## Methods

### Study design

Regional ventilation distribution was measured in seven rats in each of four body positions (prone, supine, right- and left-lateral) in random order with all He3 MRI images obtained first, followed by EIT.

### Animal preparation

The study was approved by the institutional ethics committee. Seven Wistar rats (8 to 10 weeks of age, 286 ± 21 g of either sex) were studied. The rats were anaesthetized, incubated and prepared as previously described [8]. The chest was circumferentially shaved and 16 epicardial pacing wires (Medtronic Inc, Minneapolis, MN, USA) were sutured through the skin and the panniculosus carnosus with equal inter-electrode spacing [8].

The rats were ventilated using a MRI compatible time-cycled, pressure-limited ventilator based on that of Hedlund [9] with a respiratory rate of 80 breaths per minute and a tidal volume of ~10 mL/kg.

### Hyperpolarized helium MRI (He3 MRI)

Rats were imaged with a Bruker (Ettlingen, Germany) 4.6 T ADVANCE spectrometer running Paravision 5.0 software. A 72 mm ID volume coil containing a ^1^ H (190.2 MHz) and ^3^He (144.9 MHz) RF coils was used. A single 40 mm ^3^He axial projection was acquired through the lungs with the following parameters: TR = 6 ms, TE = 1.7 ms, excitation pulse angle = 22°, field of view = 40 × 40 mm, acquisition matrix 96 × 60, centric phase encoding, scan time = 360 ms. The data was zero filled to give an image matrix of 128 × 88.

Three 2 ml breaths of He3 MRI were used to wash air from the lungs and increase the signal-to-noise ratio of the final image. Following washout, a 2 ml breath of HP3He was given with an inspiratory pause of 2 seconds. The images were acquired during this pause. The rat’s position was then changed and ventilation continued with air for at least 1 minute before the next He3 MRI image. The pacing wires used for EIT were removed prior to MR scanning but the scan position was adjusted to the same level on the chest as the EIT measurements were taken from. Switching of the inhaled tracer gas was achieved with computer controlled mechanical valves and gated to the MRI and it was not necessary to disconnect the animal from the ventilator.

### Electrical impedance tomography (EIT)

A Göttingen GoeMF II EIT tomography (Sensor medics/VIASYS Healthcare, Netherlands) was used [10]. For the EIT measurements, the rats were ventilated with 70% helium with 30% oxygen (Heliox). Measurements were taken at 44 frames per second with a 100 kHz, 5mA_pk-pk_ current. Data were processed as previously described [6].

### Data analysis

#### Image processing

The 128 × 88 pixel He3 MRI images were scaled [11] to 1408 × 1408 pixels (lowest common multiple) in order to make individual pixels square, consistent with the EIT data. This scaling did not alter the aspect ratio of the image. The 32 × 32 pixel EIT images were scaled [11] to 1408 × 1408 pixels without interpolation to produce an image size consistent with that of the 3He MRI. The EIT back projection algorithm assumes a round cross-section of the chest, which is not the case in rodents. The chest circumference of rodents has a relatively prominent sternum but the lungs within have a more or less round shape. To investigate the impact of this assumption of the EIT back projection algorithm and to compare the anatomical images given by HP3He MR imaging and EIT, the anterior to posterior and the left to right diameters (in pixels) were reported. The number of non-zero pixels was determined for each image.

As both techniques produce pixel-based images, two measures of regional ventilation distribution, commonly used in the analysis of EIT images, were employed to analyze both the He3 MRI and EIT data.

The geometric centre (GC) of each image was calculated using the “weighted centre of mass” function available in Imaged (free image analysis software provided by NIH) [10]. The GC is a measure for ventilation distribution and identifies preferentially ventilated lung regions. This method is computationally equivalent to previously used methods [12]. To allow comparison of different sized lung images the location of the GC was expressed as the percentage of the anterior to posterior and left to right aspect of the lung.

Tidal volume distribution, as a measure of ventilation in homogeneity, was quantified using the global in homogeneity index (GI) [13]. The median value of all non-zero pixels in the image was calculated. The absolute difference between the median and each non-zero pixel was summed, and the sum normalized to the number of non-zero pixels. The lower the GI value to more homogeneous the ventilation is distributed.

#### Statistics

Two-way ANOVAs and t-tests were used as appropriate to compare parameters between positions and techniques. Data were described using mean and standard deviation. A P-value of <0.05 was considered significant. For statistical analyses GraphPad Prism pt?>3.02 (GraphPad Software, La Jolla, USA) was used. Significance was accepted at p < 0.05.

## Results

**Figure 1:**
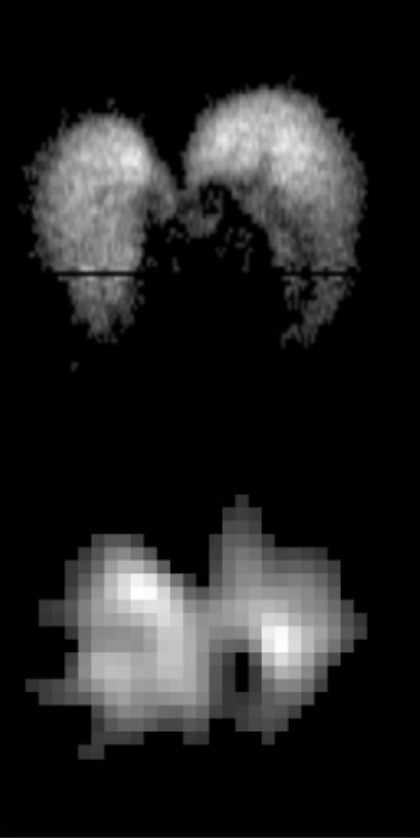
A representative He3 MRI and EIT image obtained in the same animal.

**Figure 2:**
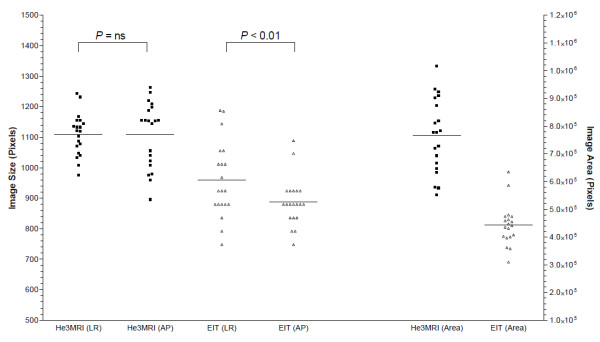
**He3 MRI and EIT image dimensions.** The diameter of each obtained image was expressed in number of pixels from anterior to posterior and from the left to right diameters and the median given in the graph. The EIT images showed a distorted image with a significant lower diameter in the AP axis whereas He3 MRI showed the same diameter for both left to right (LR) and anterior to posterior (AP) axis. EIT images were smaller than the MR images (area).

Figure 1 shows a representative image obtained with He3 MRI and EIT. Figure 2 shows the dimensions for all analyzed He3 MRI and EIT images. The He3 MRI images had the same dimensions anterior/posterior as left/right (*P* = ns). In the EIT images, the anterior/posterior dimension was smaller than the left/right (*P* < 0.01). The EIT images were smaller than the He3 MRI in both anterior/posterior and left/right dimensions (*P* < 0.01). The area of the images, as assessed by the number of non-zero pixels, was less in the EIT images (*P* <0.01).

**Figure 3:**
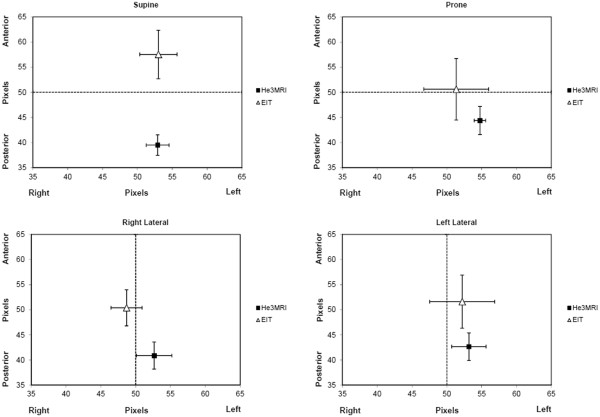
**Geometric Centre (GC) for He3 MRI and EIT images.** Differences were only analysed in the gravity axis, in supine and prone from anterior and posterior, in left and right lateral from right to left. In supine the EIT GC was significantly more anterior than the He3 MRI GC, whereas in prone no difference was found. In lateral position, the He3 MRI GC and EIT GC were not different in the right to left axis.

The locations of the GC of the EIT and He3 MRI images are shown in Figure 3. The differences in GC are only compared in the gravity axis, i.e. in supine and prone from anterior to posterior and in left and right lateral position from right to left. In supine and prone position there were significant differences in the location of the GC assessed either by EIT or He3 MRI in the anterior to posterior axis (*P* < 0.001). The GC in the anterior to posterior axis in supine were at 39.5±2.3% based on the He3 MRI images and at 57.6±5.5% based on EIT images. Similar in prone position the GC for He3 MRI images were at 44.4± 3.2% and for EIT images at 50.6± 7.0%. Within the same imaging method the body position had no significant impact on the location of the GC (P = ns). In left and right lateral position there were no differences found in the location of the GC between imaging methods and body position (analyzed in the gravity axis from right to left).

w 4 shows the calculated GI for He3 MRI and EIT images. The GI for He3 MRI measurements was 0.401±0.027 in left lateral position and for EIT 0.396±0.043 respectively, in right lateral position 0.384±0.072 and 0.363±0.014, in prone 0.370±0.046 and 0.425±0.034 and in supine position 0.401± 0.038 and 0.375± 0.011 (mean±SD). No effect of imaging technique or body position or interaction between imaging technique and position was found (*P* = ns).

**Figure 4:**
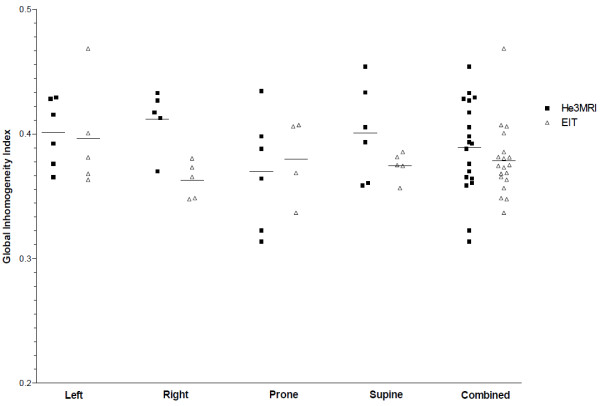
**Global Inhomogeneity Index (GI) for He3 MRI and EIT images.** There were no statistically significant differences between He3 MRI and EIT.

## Discussion

In this experimental study we measured regional ventilation distribution and ventilation in homogeneity with He3 MRI and EIT. There were significant differences for the assessment of regional ventilation distribution between the two measurement techniques but not for the assessment of global ventilation in homogeneity.

He3 MRI is an anatomical imaging modality with the ability to show gas distribution within the lung. Few comparisons of He3 MRI with other imaging modalities have been made. [14] In a previous study using EIT we have shown, that measurement of regional ventilation distribution and ventilation in homogeneity was not affected by gas density. [6] This current study was a direct comparison between He3 MRI and EIT with inhalation gases of similar density.

The location of the GC is a measure of ventilation distribution. A significant difference was measured between prone and supine positions, by both techniques. The position of the GC in the anterior/posterior plane was also significantly different between the two techniques. The reason for this is probably an artifact in the reconstruction of the EIT images. Like all MRI, He3 MRI produces an anatomically accurate image. This need not be the case with EIT. The weighted back-projection algorithm employed assumes that the electrodes are equally spaced on the circumference of a circle around the chest. Uneven spacing of the electrodes or a non-circular cross-section causes distortion in the reconstructed image. Figure 2 shows the dimensions of the He3 MRI and EIT images. The He3 MRI images show that the lungs are essentially circular in cross-section with the anterior-posterior and right-left dimensions being the same. The EIT images are smaller overall, with the anterior-posterior dimensions being smaller than the left-right. This can be explained by the anatomical shape of the outside of the rat’s thorax, where the electrodes were located, not being circular and the conductivity differences of the various tissue layers causing signals which result in relative image shrinkage [15].

The GI is a measure of ventilation in homogeneity independent of any spatial distortion in the image and the number of pixels in the image. We found that the GI was the same when measured with both He3 MRI and EIT, with no positional dependence seen. This is consistent with our previous results which showed the GI to be independent of position and gas density.

This comparison of EIT with He3 MRI has certain limitations. He3 MRI images are obtained at a steady-state, usually after several inhalation cycles of the tracer gas [2]. As such, these images are dependent on both convention and diffusion of the inhaled gas. The images are a reflection of the gas concentration in the alveolar and airway space at a given point in the respiratory cycle. EIT measurements depend on electrical impedance changes within the chest [16], and the majority of these changes occur in the airways as a result of convection dependent volume changes. There is little change in the alveolar space during tidal breathing, and these regions are only contribute to the EIT image if alveolar recruitment or de-recruitment occurs [17]. Hence EIT detects changes in ventilation distribution that are more convection, and less diffusion, dependent. Considering the high infusibility of helium, MR imaging detects lung regions that are both convection and diffusion dependent.

EIT images provide a single slice of undeterminable thickness cross-section of the chest. He3 MRI images were taken more or less of the total lung (4 × 4 cm view). Hence the He3 MRI images may include a greater contribution from the apical and caudal lung regions.

Despite fundamental differences, the two techniques were able to detect changes in lung ventilation induced by positional changes. The measure of ventilation in homogeneity was the same for both techniques. EIT and He3MRI both measure functional distribution of air whereas regular computer tomography only allows determining air filled regions that potentially may not contribute to gas exchange.

## Conclusion

Both EIT and He3 MRI show a high agreement in the assessment of ventilation distribution.

## Competing interests

The authors declare that they have no competing interests.

## Authors’ contributions

JF, GG and AS conceived the study. KD, MF and GC carried out the laboratory work and data analysis. KD and AS drafted the manuscript, to which all authors contributed and approved the final version.

## Funding

This study was funded by the National Health and Medical Research Council.
